# hTERT gene knockdown enhances response to radio- and chemotherapy in head and neck cancer cell lines through a DNA damage pathway modification

**DOI:** 10.1038/s41598-018-24503-y

**Published:** 2018-04-13

**Authors:** Wojciech Barczak, Agnieszka Sobecka, Pawel Golusinski, Michal M. Masternak, Blazej Rubis, Wiktoria M. Suchorska, Wojciech Golusinski

**Affiliations:** 1 Department of Head and Neck Surgery, Poznan University of Medical Sciences, The Greater Poland Cancer Centre, Garbary 15 Str., 61-866 Poznan, Poland; 2 Radiobiology Lab, The Greater Poland Cancer Centre, Garbary 15 Str., 61-866 Poznan, Poland; 3 Head and Neck Cancer Biology Lab, Department of Biology and Environmental Studies, Poznan University of Medical Sciences, Poznan, Poland; 4 University of Central Florida, Burnett School of Biomedical Sciences, College of Medicine, FL 32827 Orlando, USA; 5 Department of Clinical Chemistry and Molecular Diagnostics, Poznan University of Medical Sciences, Przybyszewskiego 49 Str., 60-355 Poznan, Poland; 6 Department of Electroradiology, Poznan University of Medical Sciences, Garbary 15 Str., 61-866 Poznan, Poland

## Abstract

The aim of the study was to analyze the effect of hTERT gene knockdown in HNSCC cells by using novel *in vitro* models of head and neck cancer (HNSCC), as well as improving its personalized therapy. To obtain the most efficient knockdown siRNA, shRNA-bearing lentiviral vectors were used. The efficiency of hTERT silencing was verified with qPCR, Western blot, and immunofluorescence staining. Subsequently, the type of cell death and DNA repair mechanism induction after hTERT knockdown was assessed with the same methods, followed by flow cytometry. The effect of a combined treatment with hTERT gene knockdown on Double-Strand Breaks levels was also evaluated by flow cytometry. Results showed that the designed siRNAs and shRNAs were effective in hTERT knockdown in HNSCC cells. Depending on a cell line, hTERT knockdown led to a cell cycle arrest either in phase G1 or phase S/G2. Induction of apoptosis after hTERT downregulation with siRNA was observed. Additionally, hTERT targeting with lentiviruses, followed by cytostatics administration, led to induction of apoptosis. Interestingly, an increase in Double-Strand Breaks accompanied by activation of the main DNA repair mechanism, NER, was also observed. Altogether, we conclude that hTERT knockdown significantly contributes to the efficacy of HNSCC treatment.

## Introduction

Malignant tumors of the head and neck are the sixth leading cancer worldwide, accounting for approximately 600,000 cases per year with the number of deaths reaching almost to 380,000. Among head and neck cancers, over 95% are squamous cell carcinomas, ascending from epithelial cells that line the mucosal surfaces^1^. Depending on histological diagnosis and localization, HNSCCs differentiate in terms of clinical outcome and prognosis, however the diagnostic and therapeutic problems are similar. In order to maximize radicalization of anti-tumor therapy, a combination of local treatments (surgery, radiotherapy) with chemotherapy is commonly used. Such an approach improves patients’ outcomes and increases overall survival^2^. Intensification of this effect could be obtained by an adjuvant molecular therapy. One of the most promising strategies is RNA interference targeting telomerase. However, this process still requires more advanced studies to thoroughly assess its advantages.

A crucial step in cancer development is the ability to undergo unlimited cell divisions, possible mainly due to telomerase activity restoration. It has been shown that telomerase is functional in about 90% of cancers. However, its activity is not observed in the majority of somatic cells. The strategy of cancer therapy based on telomerase regulation is currently widely used (antisense nucleotides, ribozymes, vitamin D, G-quadruplex stabilizers, adenoviral vectors)^3–5^. But due to the complexity of the process, there is still much to discover. Even if various mechanisms of cell death—including autophagy, mitotic catastrophe, and necrosis—share some common areas, it is still difficult to apply this knowledge to cancer therapy. Even targeting telomerase may appear less efficient than expected since some cancer cells can develop a telomerase-independent way of telomere restoration, i.e., Alternative Telomere Lengthening (ALT)^6^. Consequently, it is difficult to describe the associations between telomerase and cancer cell metabolism. In any case, it is difficult to transfer this knowledge into clinics.

RNA interference as an effective system for silencing gene expression has found its application in gene therapy. Given the transfection efficiency and ease of delivery, the use of siRNA is more advantageous than shRNA. Takahashi *et al*. (2009) observed higher siRNA transfection efficiency when compared to shRNA in cells with low proliferative potential^7^. Additionally, the lower molecular weight of siRNA—when compared to shRNA—makes these molecules easier to deliver to cells and viral and non-viral systems are the main methods of delivering interfering RNA particles into cells. Retroviral vectors including lentiviruses, adenoviruses, and adeno-associated viruses are especially considered as being potentially effective vectors in cancer gene therapy. However, the construction of a safe, efficient, and universal system is still a challenge^8–11^.

In this project we analyzed the efficiency and potency of RNA interference (siRNA and shRNA) directed against hTERT in order to eliminate cancer cells. And due to the complex nature of carcinogenesis processes and the contribution of many factors to the control of tumor growth, the possibility of using shRNA against telomerase may very well provide a novel approach when administrating cytostatics and/or radiation therapy. Consequently, it might lead to a reduction in drug and radiation doses, and a decrease in harmful side effects.

## Results

### Assessment of hTERT downregulation efficiency

**Figure 1:**
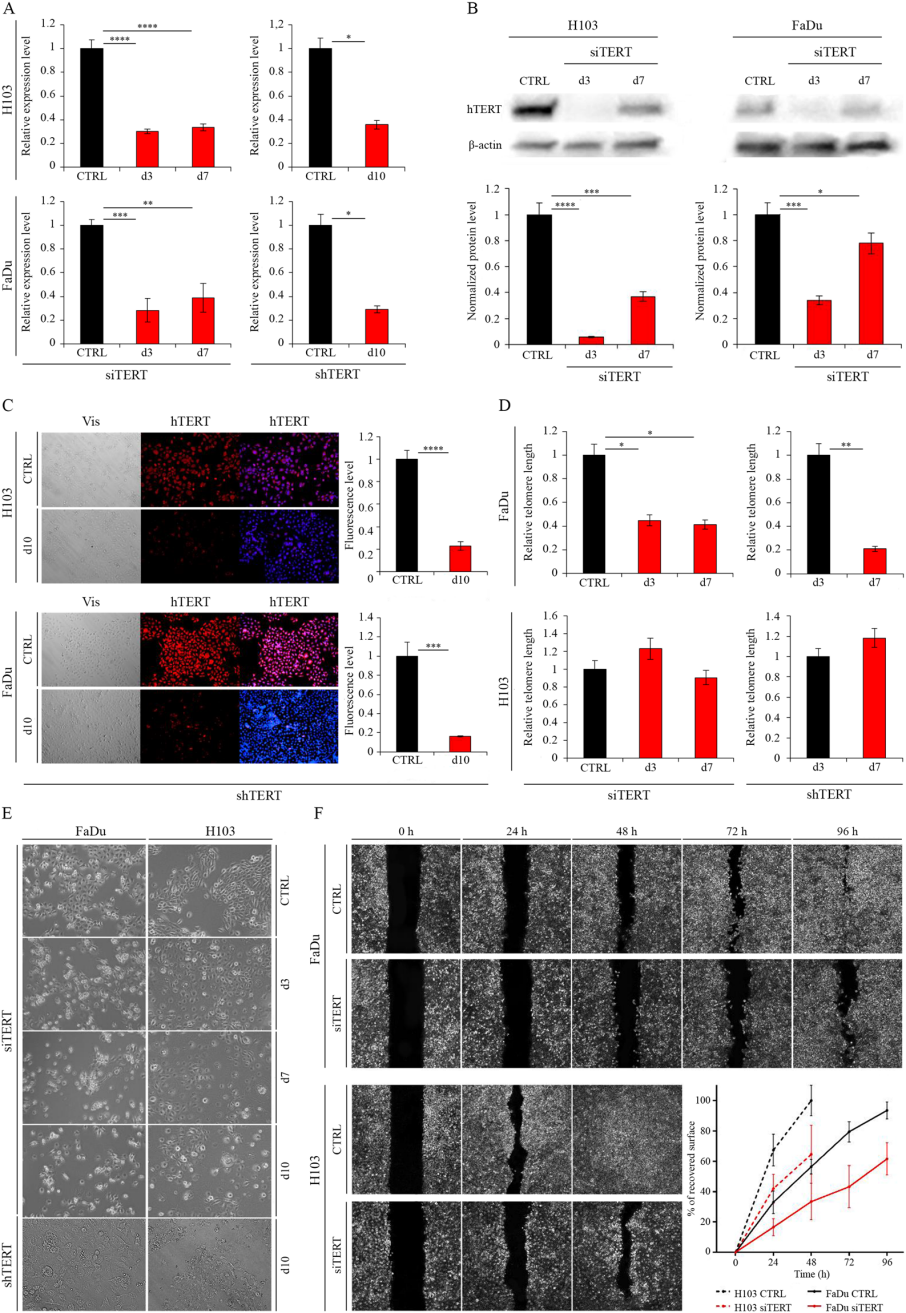
Analysis of siRNA/shRNA efficiency after hTERT gene knockdown in HNSCC cell lines. (**A**) hTERT gene expression at the transcriptional level measured by qPCR; (**B**) hTERT gene expression at the protein level (siRNA) with Western blot analysis. Semi-quantitative analysis of Western blot results using ImageJ software; (**C**) hTERT gene expression at the protein level (shRNA) by immunofluorescence staining, objective magnification 20x; (**D**) relative telomere length analysis by qPCR; (**E**) cell proliferation microscopic observation, objective magnification 20x; (**F**) cell migration wound healing assay, objective magnification 20x. CTRL – control cells transfected with non-specific siRNA/cells transduced with control lentiviral vector; siTERT – cells transfected with hTERT gene-targeted siRNA on day 3^rd^ and 7^th^; shTERT – cells transduced with lentiviral vectors containing shRNA. Data are presented as the mean ± standard deviation. *P < 0.05, **P < 0.01, ***P < 0.001, ****P < 0.0001 with comparisons indicated by lines.

Cells were subjected to an analysis of hTERT downregulation effect 72 hours after transfection. At that time a significant effect was observed in both FaDu and H103 cells at the transcriptional level (downregulation at 72%, p* = *0.0003 and 69%, p ≤ 0.0001, relative to mock siRNA, respectively) as well as at the protein level (downregulation at 66%, p = 0.0003 and 94%, p ≤ 0.0001, respectively) when immunodetection was applied. The effect was less efficient but still significant after the next 4 days (altogether 7 days) in both cell lines at the transcriptional level (61%, p* = *0.0012 and 66%, p ≤ 0.0001, respectively), as well as at the protein level (22%, p = 0.0328 and 63%, p = 0.0004, respectively) (Fig. 1A,B). hTERT knockdown was even more persistent when shRNA was applied, showing a significant effect after 10 days of lentiviral infection in both FaDu and H103 cells at the transcriptional level (downregulation at 71%; p = 0.0106 and 64%; p = 0.0135, respectively) (qPCR) and protein level (downregulation at 84%; p = 0.0005 and H103 − 77%; p ≤ 0.0001, respectively) (immunofluorescence staining) (Fig. 1A,C).

Noteworthy, a significant telomere attrition was detected (qPCR) in the FaDu cell line after hTERT knockdown was conducted via siRNA (59%, p = 0.0134) and shRNA (79%, p = 0.0083) when compared to control samples. Interestingly, no telomere length alteration was observed in H103 cells (Fig. 1D). Furthermore, a significant depletion of a proliferation rate was observed in FaDu and H103 cells (Fig. 1E) when a microscopic observation was applied. Moreover, cell migration ability decreased significantly after hTERT knockdown with siRNA (wound healing assay). At different time intervals in both FaDu (p = 0.037 for 24 hours and 48 hours, p = 0.015 and p = 0.009 for 72 hours and 96 hours respectively) and H103 (p = 0.036 for 24 hours and p = 0.046 for 48 hours, respectively) migration potential decreased when assessed with a wound healing test (Fig. 1F).

### Cell cycle arrest analysis

**Figure 2:**
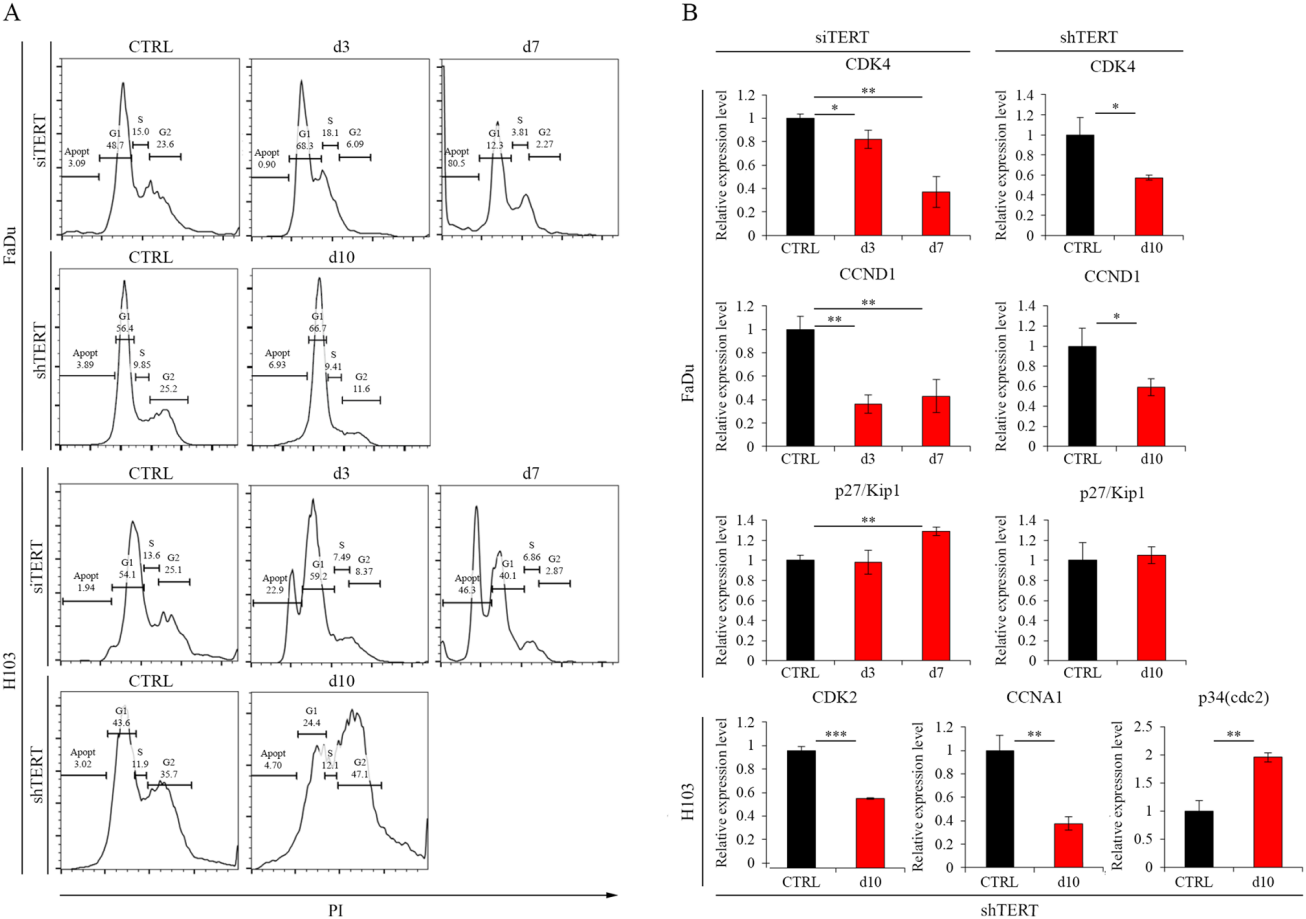
Cell cycle arrest analysis after silencing of hTERT gene expression. (**A**) Flow cytometry analysis; (**B**) Analysis of cell cycle arrest-related genes expression at the transcriptional level by qPCR. CTRL – control cells transfected with non-specific siRNA/cells transduced with control lentiviral vector; siTERT – cells transfected with hTERT gene-targeted siRNA on day 3^rd^ and 7^th^; shTERT – cells transduced with lentiviral vectors containing shRNA on day 10^th^. Data are presented as the mean ± standard deviation. *P < 0.05, **P < 0.01, ***P < 0.001, ****P < 0.0001 with comparisons indicated by lines.

In order to analyze the impact of hTERT downregulation on the cell cycle, a flow cytometry analysis using propidium iodide was performed. In both cell lines, a significant increase in the apoptotic cell fraction was observed when compared to the control sample. In the FaDu cell line, on day 7, a fraction of the apoptotic cells reached 75.8%. In the control, however, it was barely detectable (3.23%). In H103 cells, distribution of fractions was not that vivid; apoptotic cells reached 47.9% compared to control (2.68%). Three days after transfection in the H103 cells, apoptotic fraction was increased to 22.9%. At the same time point in the FaDu cell line, elevated G1 fraction (from 46.6% to 62.2%) with simultaneous depletion of G2 fraction (from 28.1% to 13.1%) was observed, indicating a cell cycle arrest in G1 phase (Fig. 2A). In case of hTERT gene silencing with shRNA, an increased percentage of G1 phase (10.3% more cells compared to control) and a decreased percentage of cells in G2 (13.6%) was also noticed. In H103 cells, an increase in the fraction of cells in G2 phase (11.4%) and a decrease in the G1 phase (19.2%) has been shown (Fig. 2A).

In order to confirm the cell cycle arrest in the G1 phase after hTERT gene silencing in FaDu cells, the expression of markers of the above phase are the following: CDK4, CCND1, and p27/Kip1 which were assessed with qPCR. A decrease in CDK4 (day 3, 82%, p = 0.023, day 7, 37% p = 0.0013) and CCND1 (day 3, 36%, p = 0.0012; day 7, 43%, p = 0.0052) gene expression at days 3 and 7 was demonstrated. In addition, an increase in p27/Kip1 gene expression at day 7 was noticed (129%, p = 0.0014) (Fig. 2B). In case of the use of lentiviral vectors, we also observed a significant decrease in the expression of genes CKD4 (57%, p = 0.0232), and CCND1 (59%, p = 0.0227) in the FaDu cell line. Furthermore, we observed a decreased expression of markers of S/G2 cell cycle arrest, which are: CDK2 (59% p ≤ 0.0001) and CCNA1 (38%, p = 0.0016). We then observed an increased expression of p34/Cdc2 (195%, p ≤ 0.0001), demonstrated in H103 cells (Fig. 2B).

### Cell death assessment – flow cytometry analysis

**Figure 3:**
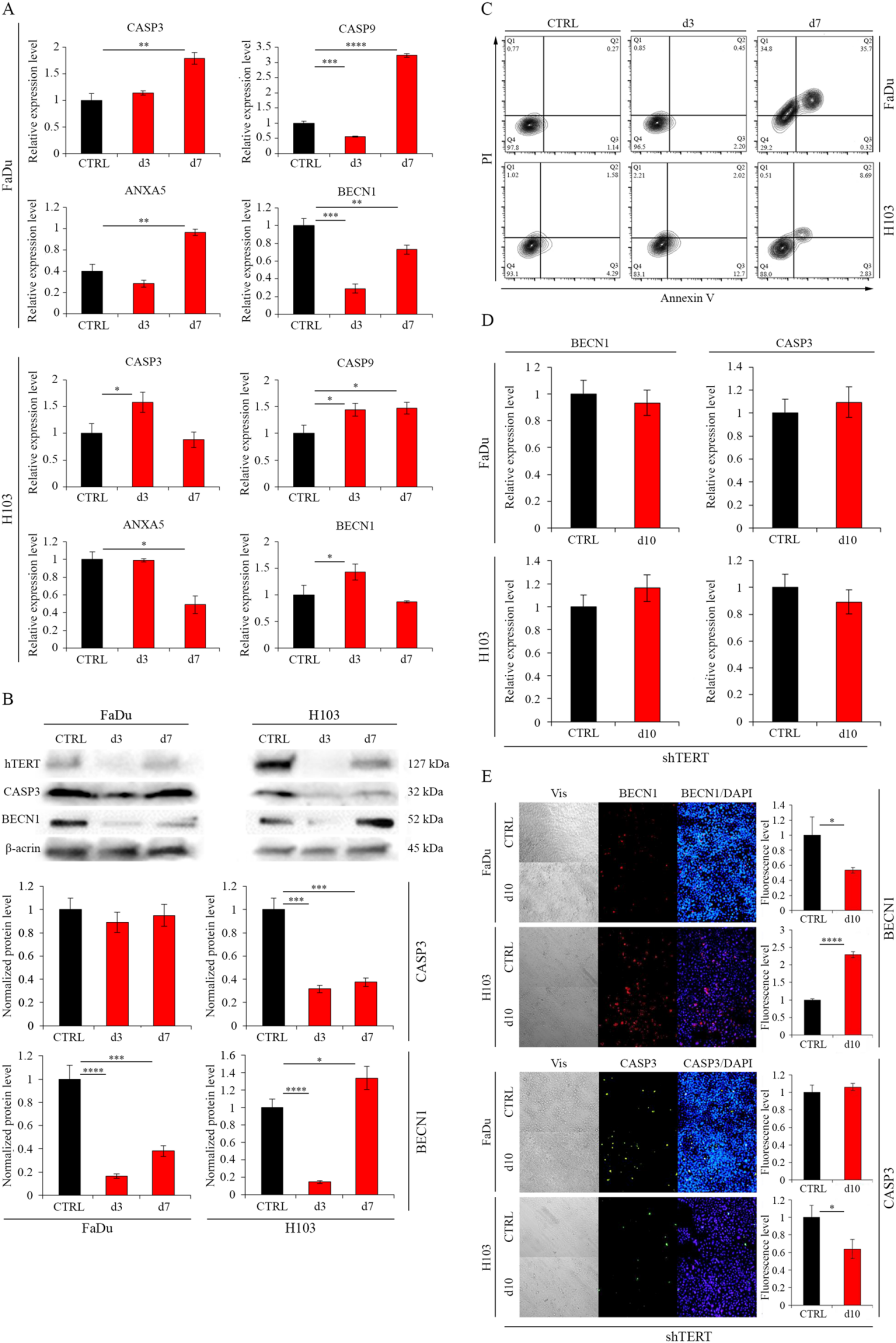
Analysis of cell death mechanism after hTERT gene knockdown in HNSCC cell lines. (**A**) siRNA - Analysis of apoptosis- and autophagy-related genes expression at the transcriptional level with qPCR; (**B**) siRNA - Analysis of apoptosis- and autophagy-related genes expression at the protein level by Western blot analysis. Semi-quantitative analysis of Western blot results using ImageJ software; (**C**) Apoptosis analysis by flow cytometry; (**D**) shRNA - Analysis of apoptosis- and autophagy-related genes expression at the transcriptional level with qPCR; (**E**) shRNA - Analysis of apoptosis- and autophagy-related genes expression at the protein level by immunofluorescence staining, objective magnification 20x. CTRL – control cells transfected with non-specific siRNA/cells transduced with control lentiviral vector; siTERT – cells transfected with hTERT gene-targeted siRNA on day 3^rd^ and 7^th^; shTERT – cells transduced with lentiviral vectors containing shRNA on day 10^th^. Data are presented as the mean ± standard deviation. *P < 0.05, **P < 0.01, ***P < 0.001, ****P < 0.0001 with comparisons indicated by lines.

Due to the demonstrated effect of hTERT gene silencing on cell cycle arrest and the ambiguous literature data suggesting the effect of hTERT gene silencing on apoptosis induction), an attempt to verify this theory was made. In both FaDu and H103 cell lines, an increased level of apoptosis markers was observed: in FaDu cells upregulation of CASP3, CASP9, and ANXA5 (179% p = 0.0012; – 323% p ≤ 0.0001; – 191% p = 0.001, respectively) at day 7. Also, in H103 cells, an elevated level of apoptosis markers was noticed 3 days after transfection (CASP3 − 158%, p* = *0.0143; CASP9 – 144%, p* = *0.0119) when qPCR and Western blot analyzes were performed. The effect was most significant 7 days after transfection. Moreover, on day 7 the increased expression of BECN1 was observed in H103 cells (BECN1 – 143%, p* = *0.0336) (Fig. 3A,B). Activation of apoptosis after hTERT knockdown was confirmed by flow cytometry with Annexin V and propidium iodide staining. An elevated fraction of apoptotic cells was demonstrated 7 days after transfection in cell line FaDu (from 0.27% to 35.7%; p < 0.0001) and cell line H103 (from 1.58% to 8.69%; p < 0.0002). In the case of FaDu cells, propidium iodide-positive necrotic fraction on the 7^th^ day was also observed (from 0.77% to 34.8%; p < 0.0001) (Fig. 3C).

Importantly, knockdown of hTERT via lentiviral vector did not cause activation of apoptosis as observed in the experiment with FaDu and H103 cell lines. However, an increased level of BECN1 was observed in H103 cells (229%, p ≤ 0.0001, relative to control) (Fig. 3D,E).

### Induction of apoptosis after hTERT knockdown with cytostatics co-administration

**Figure 4:**
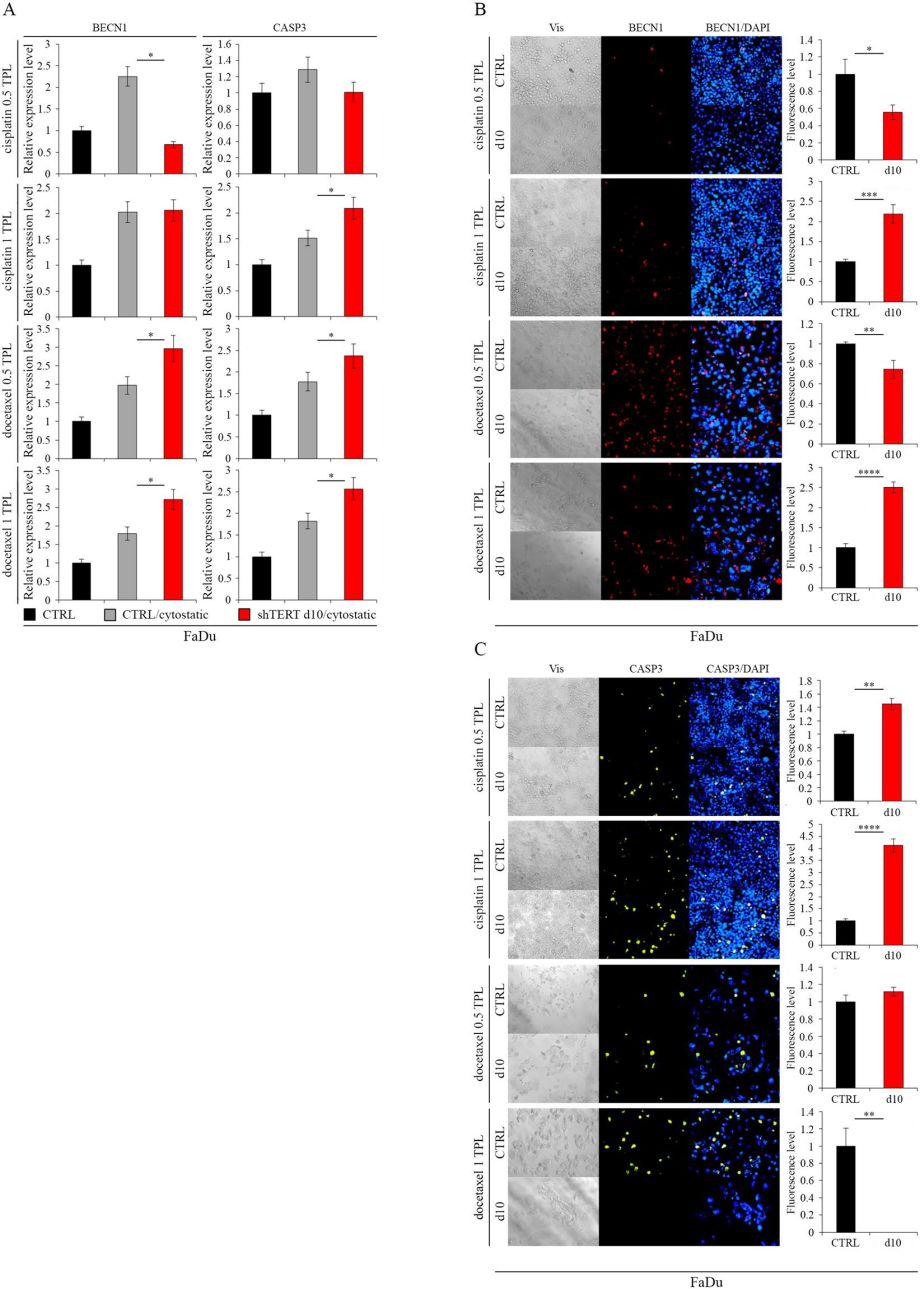
Induction of apoptosis in FaDu cells after hTERT knockdown (lentiviral vectors) with cytostatics co-administration. (**A**) Analysis of apoptosis- and autophagy-related genes expression at the transcriptional level by qPCR; (**B**,**C**) Analysis of apoptosis- and autophagy-related genes expression at the protein level with immunofluorescence staining, objective magnification 20x. Cisplatin – 0.5 TPL (3.33 μM) and 1 TPL (6.67 μM); docetaxel – 0.5 TPL (275 nM) and 1 TPL (550 nM). CTRL - cells transduced with control lentiviral vector; shTERT d10/d10 - cells transduced with lentiviral vectors containing shRNA on day 10^th^. Data are presented as the mean ± standard deviation. *P < 0.05, **P < 0.01, ***P < 0.001, ****P < 0.0001 with comparisons indicated by lines.

Studied cells were more susceptible to death when cytostatic compounds were combined with hTERT downregulation. In FaDu cells, however, the expression of BECN1—measured with qPCR at the transcription level—was elevated following docetaxel administration at 0.5 TPL, i.e., 275 nM (p = 0.0193) and 1 TPL, i.e., 550 nM (p = 0.0182) concentrations. CASP3 gene expression at 1 TPL of cisplatin (p = 0.0341) and docetaxel at 0.5 TPL (p = 0.0418) and 1 TPL (p = 0.0282) was also increased (Fig. 4A). A significant effect of hTERT downregulation and cytostatics administration on gene expression analyzed at the protein level was also noted. We observed an increase in BECN1 accumulation (IF assay) after administering cisplatin and docetaxel at a concentration of 1 TPL (p = 0.0009; p ≤ 0.0001 respectively) (Fig. 4B). A significant increase in the CASP3 level was also revealed after cisplatin administration (0.5 TPL [p = 0.0011] and 1 TPL [p ≤ 0.0001]) (Fig. 4C).

**Figure 5:**
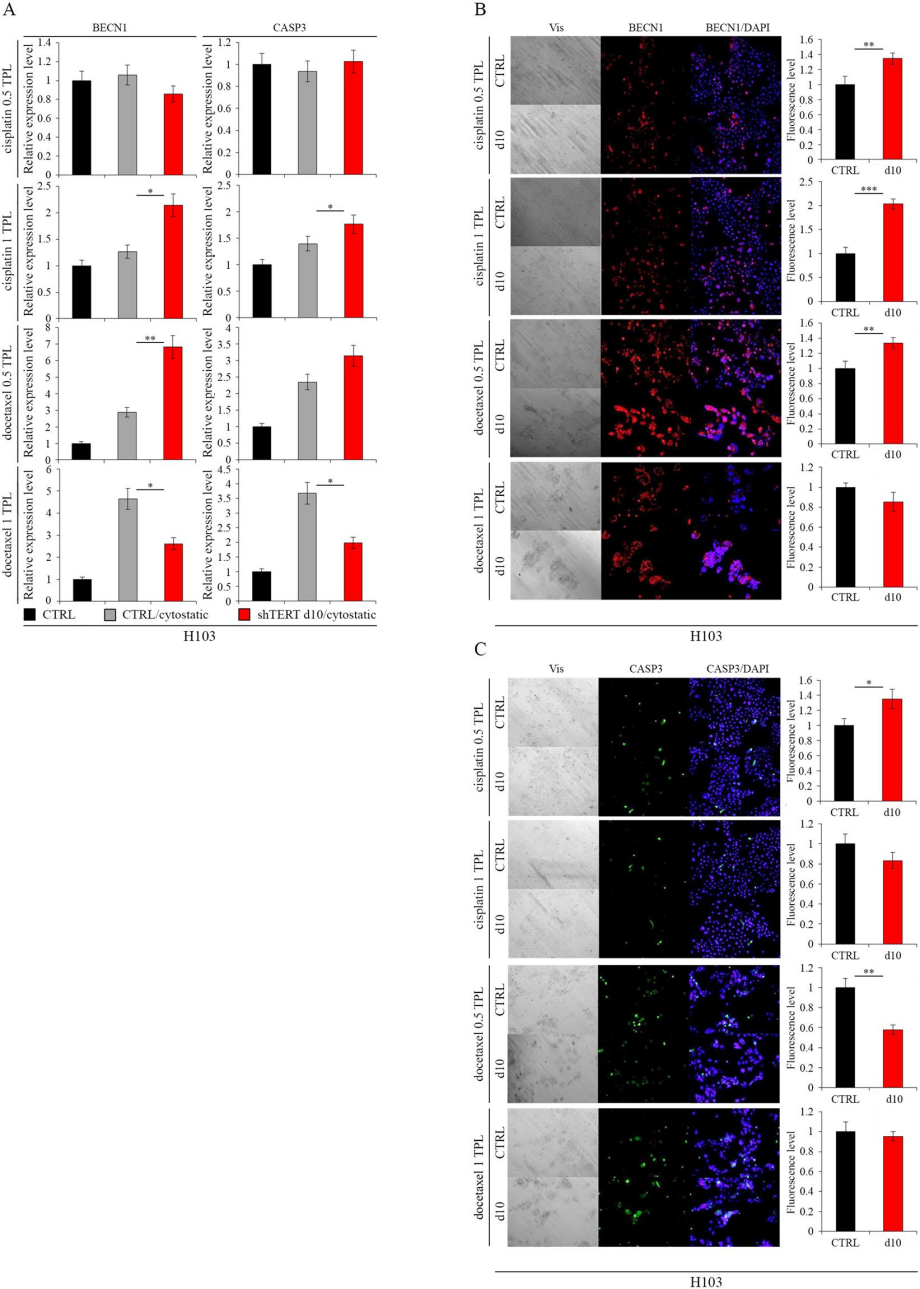
Induction of apoptosis in H103 cells after hTERT knockdown (lentiviral vectors) with cytostatics co-administration. (**A**) Analysis of apoptosis- and autophagy-related genes expression at the transcriptional level by qPCR; (**B**,**C**) Analysis of apoptosis- and autophagy-related genes expression at the protein level with immunofluorescence staining, objective magnification 20x. Cisplatin – 0.5 TPL (3.33 μM) and 1 TPL (6.67 μM); docetaxel – 0.5 TPL (275 nM) and 1 TPL (550 nM). CTRL - cells transduced with control lentiviral vector; shTERT d10/d10 - cells transduced with lentiviral vectors containing shRNA on day 10^th^. Data are presented as the mean ± standard deviation. *P < 0.05, **P < 0.01, ***P < 0.001, ****P < 0.0001 with comparisons indicated by lines.

In H103 cells, expression of BECN1 at the transcription level was elevated after cisplatin was administered at doses of 1 TPL, i.e. 6.67 µM (p = 0.0372) and docetaxel at 0.5 TPL (p = 0.0098). There was an increase in gene expression of CASP3 after administering cisplatin at a concentration of 1 TPL (p = 0.0443) (Fig. 5A). Changes in expression of the BECN1 and CASP3 genes were also observed at the protein level. An increased expression of the BECN1 gene after cisplatin (0.5 TPL [p = 0.0085], 1 TPL [p = 0.0005]) and docetaxel (0.5 TPL [p = 0.0074]) administration was detected (Fig. 5B), while CASP3 levels were elevated after administering cisplatin (0.5 TPL [p = 0.0153]) (Fig. 5C).

### The effect of the hTERT gene silencing on Double-Strand Breaks after irradiation alone and with concomitant use of cisplatin- and docetaxel-based chemotherapy

To investigate the effect of the hTERT gene silencing on Double-Strand Breaks (DSBs) of DNA in FaDu and H103 cell lines, cells were irradiated with three different doses (0.5, 1, and 2 Gy), followed by cytometric analysis of DSBs marker - γH2AX.

**Figure 6:**
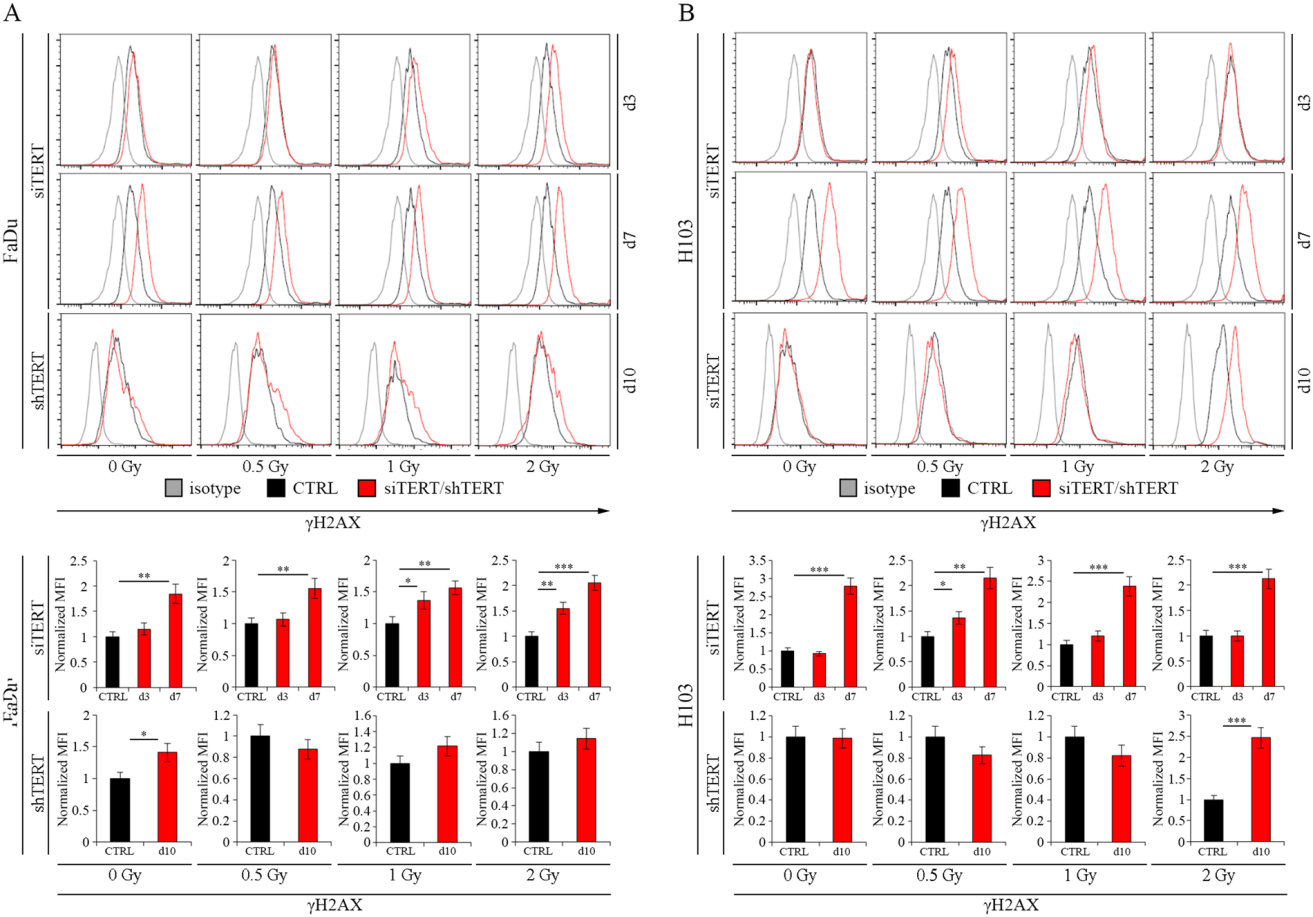
The impact of hTERT gene downregulation on Double-Strand Breaks level after ionizing radiation alone in HNSCC cell lines. (**A**) cytometric analysis of Double-Strand Breaks marker - γH2AX – FaDu cells; (**B**) cytometric analysis of Double-Strand Breaks marker - γH2AX – H103 cells. CTRL – control cells transfected with non-specific siRNA/cells transduced with control lentiviral vector; siTERT – cells transfected with hTERT gene-targeted siRNA on day 3^rd^ and 7^th^; shTERT – cells transduced with lentiviral vectors containing shRNA on day 10^th^. Data are presented as the median ± standard deviation. *P < 0.05, **P < 0.01, ***P < 0.001, ****P < 0.0001 with comparisons indicated by lines.

We found that FaDu cells with hTERT knockdown present an increased level of γH2AX when compared to control after irradiation with 1 and 2 Gy dose on day 3 (1.36 MFI, p = 0.0223 and 1.55 MFI, p = 0.0037, respectively). On day 7, the DSBs level was higher, regardless of radiation dose: 0 Gy – 1.85 MFI (p = 0.0024), 0.5 Gy – 1.55 MFI (p = 0.0072), 1 Gy – 1.56 MFI (p = 0.0028), 2 Gy – 2.05 MFI (p = 0.0005). Elevated levels of DSBs after hTERT silencing with shRNA were only noticed after gene expression depletion (1.41 MFI, p = 0.0145) (Fig. 6A). A similar observation was performed in the H103 cell line, where an increased level of DSBs on day 3 was demonstrated after irradiation of 0.5 Gy (1.37 MFI, p = 0.0191), and on day 7 regardless of radiation dose (0 Gy – 2.79 MFI [p = 0.0002], 0.5 Gy – 2.15 MFI [p = 0.0011], 1 Gy – 2.38 MFI [p = 0.0007], 2 Gy – 2.13 MFI [p = 0.0008]). In case of usage of lentiviral vectors, elevated DSBs levels were observed after 2 Gy (2.48 MFI, p = 0.0007) (Fig. 6B).

**Figure 7:**
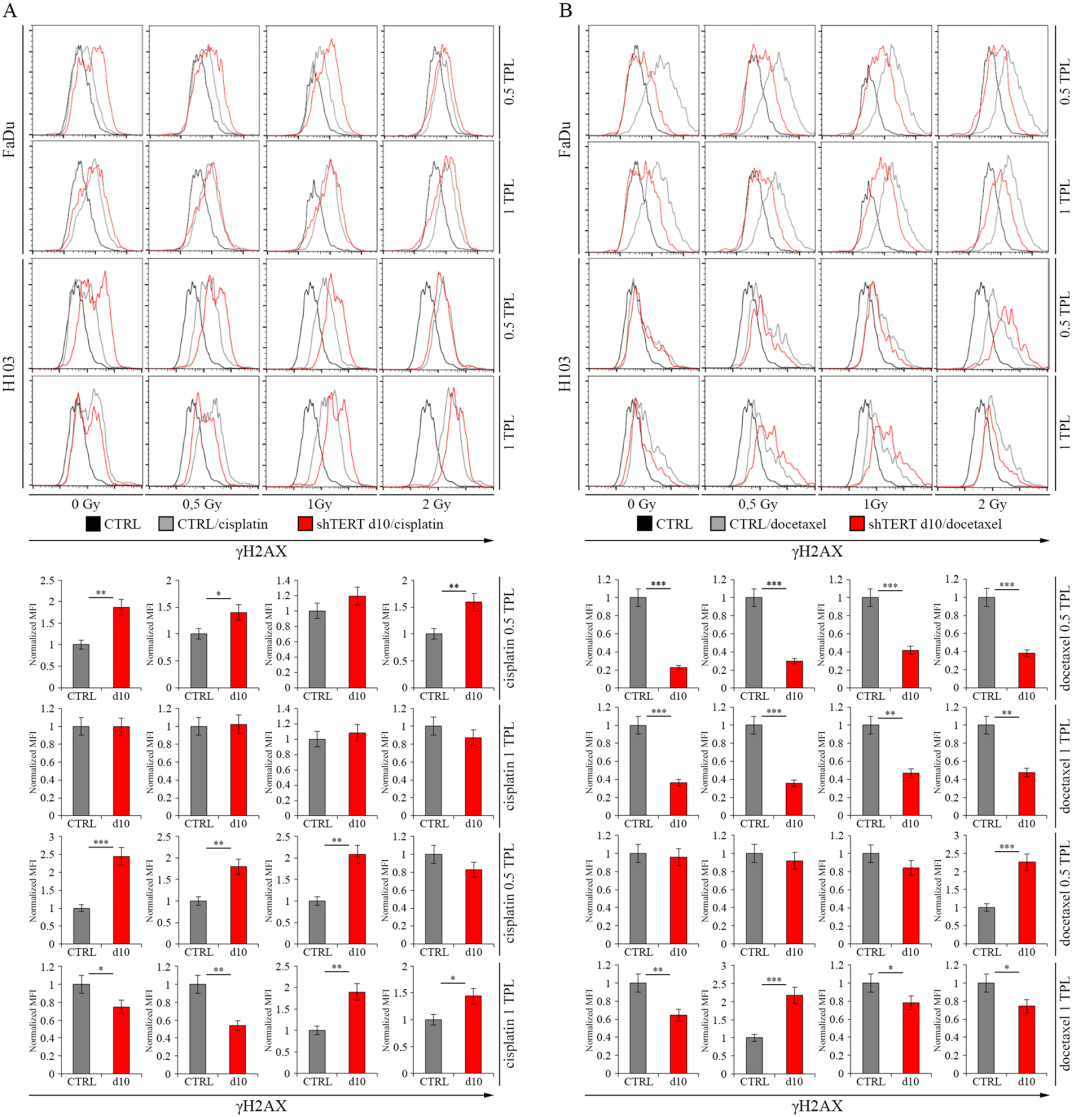
The impact of hTERT gene downregulation on Double-Strand Breaks level after ionizing radiation with concomitant use of cisplatin- and docetaxel-based chemotherapy in HNSCC cell lines. (**A**) cytometric analysis of Double-Strand Breaks marker - γH2AX – cisplatin; (**B**) cytometric analysis of Double-Strand Breaks marker - γH2AX – docetaxel. Cisplatin – 0.5 TPL (3.33 μM) and 1 TPL (6.67 μM); docetaxel – 0.5 TPL (275 nM) and 1 TPL (550 nM). CTRL - cells transduced with control lentiviral vector; shTERT – cells transduced with lentiviral vectors containing shRNA on day 10^th^. Data are presented as the median ± standard deviation. *P < 0.05, **P < 0.01, ***P < 0.001, ****P < 0.0001 with comparisons indicated by lines.

Furthermore, measurement of the γH2AX level in hTERT knockdown cells exposed to ionizing radiation and cytostatics (cisplatin and docetaxel [0.5 and 1 TPL]) was performed for shRNA variant. In FaDu cells, an increased level of DSBs was demonstrated after administrating 0.5 TPL cisplatin alone without irradiation (0 Gy − 1.87 MFI, p = 0.0022) and with a dose of 0.5 Gy (1.40 MFI, p = 0.0158) and 2 Gy (1.59 MFI, p = 0.0055). In case of H103 cells, elevated levels of DSBs were noticed after the addition of 0.5 TPL cisplatin without irradiation (2.45 MFI, p = 0.0007) and radiation exposure of 0.5 Gy (1.8 MFI, p = 0.0026) and 1 Gy (2.09 MFI, p = 0.0012). Moreover, we observed an elevated DSBs level after 1 TPL cisplatin administration with 0.5 Gy (0.54 MFI, p = 0.0022) and without irradiation (0.74 MFI, p = 0.0244) (Fig. 7A).

The use of docetaxel led to higher DSBs levels noticed in H103 cells after irradiation with 2 Gy (0.5 TPL, 2.26 MFI, p = 0.0009) and 0.5 Gy incubated previously with 1 TPL docetaxel (2.18 MFI, p = 0.0002). In the case of FaDu cell line, concomitant hTERT knockdown and docetaxel administration resulted in a sharp rise in mortality making the analysis impossible to perform (Fig. 7B).

### DNA Damage Repair mechanisms activation

The demonstrated effect of hTERT gene silencing on cell cycle arrest and apoptosis induction and DSBs production suggested the possibility of activation of individual DNA repair mechanisms (measured with qPCR and Western blot analyzes).

**Figure 8:**
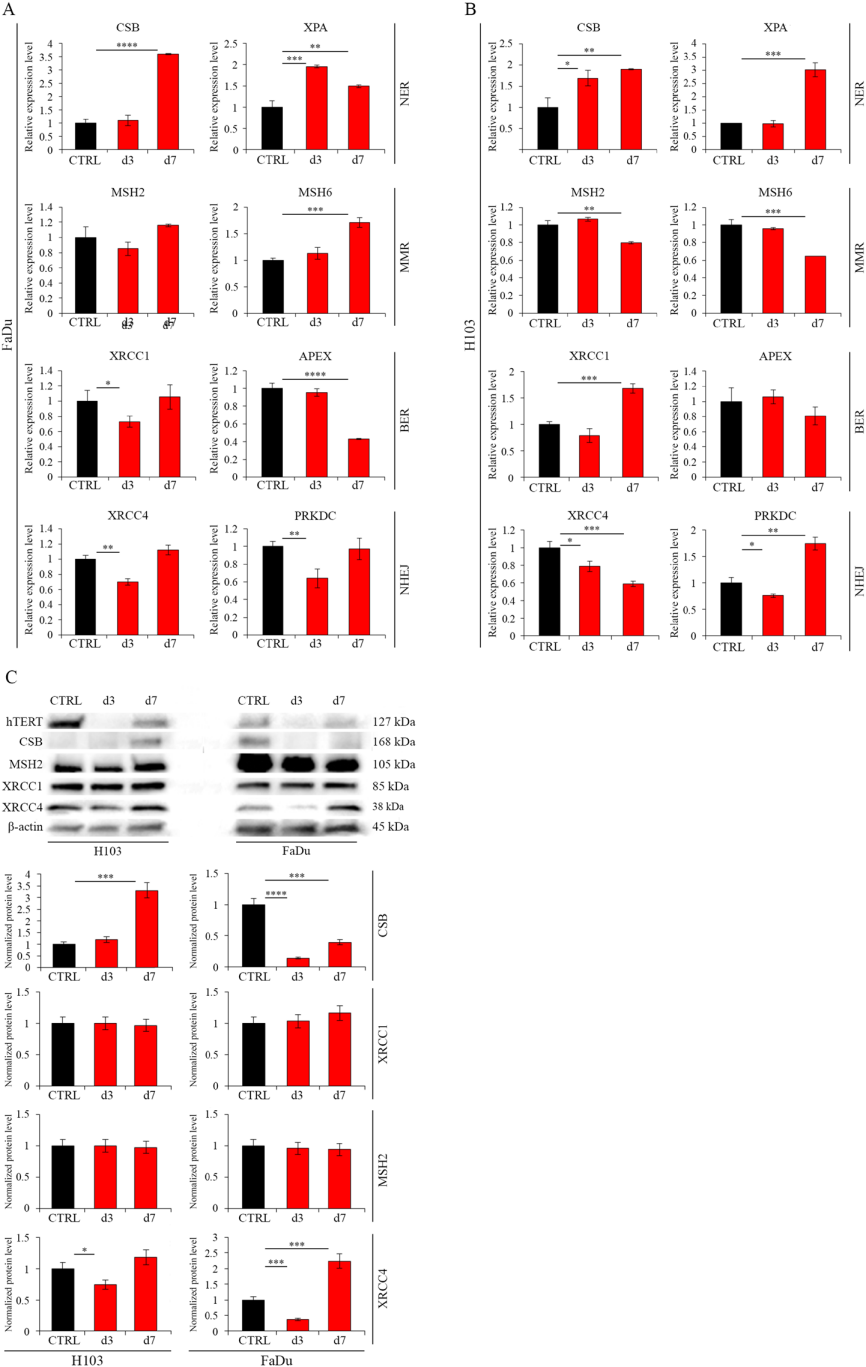
DNA Damage Repair mechanisms activation after hTERT knockdown (siRNA) in HNSCC cell lines. (**A**) Analysis of DDR-related genes expression at the transcriptional level in FaDu cells by qPCR; (**B**) Analysis of DDR-related genes expression at the transcriptional level in H103 cells by qPCR; (**C**) Analysis of DDR-related genes expression at the protein level by Western blot analysis. Semi-quantitative analysis of Western blot results using ImageJ software. CTRL – control cells transfected with non-specific siRNA; d3/d7 - cells transfected with hTERT gene-targeted siRNA on day 3^rd^ and 7^th^ respectively. Data are presented as the mean ± standard deviation. *P < 0.05, **P < 0.01, ***P < 0.001, ****P < 0.0001 with comparisons indicated by lines.

Significant increases at the transcriptional level of expression of both NER-related genes CSB (day 7^th^, 360%, p ≤ 0.0001) and XPA (day 3, 196%, p = 0.0004; day 7, 150%, p = 0.0049) was demonstrated in FaDu cells. An increased MSH6 (MMR mechanism) expression on day 7 (171%, p = 0.0002) was also noted (Fig. 8A). In the H103 cell line, a significant increase in the expression of genes involved in NER mechanism CSB (day 3, 169%, p = 0.0136; day 7, 190%, p = 0.0021) and XPA (day 7, 301%, p = 0.0002) was also shown. There was also an increase in expression of the XRCC1 (BER) (168%, p = 0.0003) and PRKDC (NHEJ mechanism) (174%, p = 0.0012) genes on day 7 (Fig. 8B).

Analysis of the protein level showed an increase in expression of the CSB gene (331%, p = 0.0003) (NER) in H103 cells at day 7 after transfection with siRNA. We also observed an elevated expression of the XRCC4 gene (224%, p = 0.0009) (NHEJ mechanism) on day 7. No increase in gene expression was detected concerning markers of the remaining DNA damage repair mechanisms (Fig. 8C).

## Discussion

The primary aim of the study was to analyze the effect of hTERT gene knockdown on cancer cell metabolism in order to develop a personalized therapy of head and neck squamous cell carcinoma. The first part of the study focused on an efficient hTERT gene expression silencing. To obtain the most effective knockdown, cells were transfected with siRNA or transduced with shRNA-bearing lentiviral vectors. Subsequently, the types of cell death and DNA repair mechanisms activated after hTERT knockdown were assessed. The ability of establishing an HNSCC cell line after hTERT gene knockdown in order to create a model for assessing the effect of a combined treatment (chemotherapy and/or radiotherapy) was also evaluated.

The use of RNA interference (shRNA or siRNA) as a tool for hTERT gene silencing has been used in several types of cancer^12–17^. In the case of head and neck cancer, only a few studies have shown a significant therapeutic potential of hTERT silencing using RNA interference, thus revealing its impact on cell death and growth^18,19^. There is also a limited number of studies where lentiviral vectors were used as a tool to carry shRNA for stable silencing telomerase expression in head and neck cancer. When Yao *et al*. (2011) investigated the effect of hTERT gene silencing by shRNA in a murine model of nasopharyngeal cancer, they demonstrated its inhibitory result on cell growth. Moreover, the influence of hTERT gene knockdown on proliferation, migration, and cell invasion inhibition was observed^20^. Also, the effectiveness of the lentiviral vectors carrying shRNA for hTERT gene expression depletion in a KB cell line, apoptosis activation and cell cycle arrest lead to the inhibition of cell proliferation, as demonstrated by Chen *et al*.^21^.

In this paper, silencing of hTERT gene encoding the telomerase catalytic subunit in HNSCC cells using siRNAs and shRNAs was performed. In the case of shRNA, lentiviral vectors were used as a carrier. For this purpose, a new shRNA molecule directed against the hTERT gene was designed and cloned into lentiviral vector pLV-THEM-GP1. A lentivector obtained this way (pLV-THEM-shTERT) has a strong H1 promoter that ensures high and stable shRNA expression.

In order to verify the functionality and efficiency of the hTERT silencing system, wide range tests were performed in FaDu and H103 established HNSCC cell lines. The analysis was made on day 3 and 7 after siRNA transfection, and on day 10 after transduction with lentiviral vectors.

The studies showed a significant reduction in hTERT gene expression at the transcriptional and protein levels in both cell lines using lentiviruses and siRNA. However, in the case of siRNA transfected cells, gene expression was restored to original levels on day 7 of the experiment. In order to confirm the efficiency of the designed sequence and to reveal the loss of telomerase catalytic function, the telomere length was measured. Both studied methods (siRNA/shRNA) successfully led to a significant shortening of telomeres in FaDu cell line. Moreover, in the case of the transfection with siRNA, this effect was observed as early as 3 days after the initial treatment. On the other hand, telomeres in H103 cell line were fully maintained after treatment with either lentiviruses or siRNA molecules, which may indicate the activation of an alternative lengthening of telomeres mechanism. The confirmation of proper functioning siRNA and shRNA was also the proliferation and migration inhibition of hTERT knockdown cells. Obtained results demonstrated the functionality of designed constructs (siRNA/shRNA).

The literature data thoroughly describing the effect of hTERT gene silencing using either siRNA or lentiviral vectors as shRNA carriers for head and neck cancer are limited, and do not indicate ambiguous molecular effects of the hTERT gene knockdown. Therefore, we decided to assess the type of cell death and DNA repair mechanisms activated following hTERT gene silencing in HNSCC cells. Furthermore, this study focuses on hTERT’s knockdown ability to alter the HNSCC cell lines response to combined treatment (chemotherapy and/or radiotherapy).

Presented literature data concerning different types of cancer indicate the activation of apoptosis after hTERT gene silencing achieved by either siRNA or shRNA. As far as head and neck cancer is concerned, we still lack data that support such a notion. Zhou *et al*. (2006) demonstrated the activation of apoptosis and the inhibition of tumor growth after hTERT knockdown using shRNA in laryngeal cancer murine model^22^. Induction of apoptosis was also observed by both, Chen *et al*. (2005, 2006)^23,24^ and Liu *et al*. (2006) in laryngeal cancer *in vitro* model^25^. Similarly, Lai *et al*. (2012) analyzed the effect of siRNA in the nasopharynx cancer *in vitro*, which indicated apoptosis is an activated type of cell death^26^. The effect of siRNA when directed against hTERT on the chemosensitivity of breast cancer cells was studied by Dong *et al*. (2009). When cells with downregulated hTERT gene expression are incubated with doxorubicin and then are compared to control cells treated with cytostatics alone, an increase in apoptotic fraction was observed^27^. Similar findings have been obtained by Guo *et al*. (2008) in the liver cancer model, where he showed increased cisplatin sensitivity following hTERT silencing with siRNA, and by Kraemer *et al*. (2006) in the bladder cancer model^28,29^. In the case of head and neck cancer, Zhao *et al*. (2015) also reported an increase in cell sensitivity to cisplatin after hTERT knockdown in the oral cancer *in vitro* model^30^.

To evaluate the effect of hTERT knockdown using the novel *in vitro* head and neck cancer model, cell death mechanism and cell cycle analysis were performed.

Due to the limited number and inconsistent literature data, we further studied the degree of apoptosis activation following the hTERT gene silencing and use of standard chemotherapeutics of head and neck cancer treatment (cisplatin and docetaxel). The analysis of gene expression—which are markers for these mechanisms—was carried out. In the case of apoptosis, expression levels of CASP3, CASP9, and ANXA5 genes were evaluated, whereas measurement of BECN1 expression was conducted as an autophagy-related gene.

When silencing the hTERT gene with siRNA, a significant increase in expression of the apoptosis markers CASP3, CASP9, and ANXA5 was shown at the transcriptional level on day 7. However, no changes were noted on day 3 except for the CASP9 gene. Decrease in BECN1 gene expression on days 3 and 7 at both the transcriptional and protein levels was also observed. In the H103 cell line, gene expression of CASP3 and CASP9 on day 3 and CASP9 gene on day 7 was observed to have magnified apoptosis induction. An increase in accumulation of the BECN1 gene at the protein level on day 7 was also demonstrated. In order to confirm the results indicating apoptosis activation, flow cytometric analysis was performed. An increase in late-stage apoptotic cells in both FaDu and H103 cell lines on day 7 was noticed. Moreover, in FaDu cells, the appearance of the necrotic fraction was observed. These results suggest that the silencing of hTERT gene expression using siRNA leads directly to the induction of apoptosis. When lentiviral vectors are used, we examined not only activation of apoptotic mechanism after the hTERT gene silencing alone, but also with the concomitant administration of cytostatics. This analysis was not possible to carry out with transfection of siRNA due to the high cell mortality. The results obtained in this study showed no activation of apoptosis after hTERT gene silencing using lentiviral vectors. Nevertheless, for the first time the effects of decreased expression of this gene on cell sensitivity to cisplatin and docetaxel on an *in vitro* HNSCC model were observed, as well as the induction of this cell death mechanism.

The activation of the apoptosis following hTERT knockdown suggests possible cell cycle arrest. The literature data do not clearly indicate the effect of this gene silencing on cell cycle in cancer cells. Zhong *et al*. (2010) showed an increase in G0/G1 cell fraction and a decrease in S/G2 phase in pancreatic cancer model^17^. Similar results were obtained by Xu *et al*. (2015) when examining the effect of hTERT gene silencing in a cellular model of liver cancer, as well as by Shi *et al*. (2014) in studies on cervical cancer^13,15^. Luo *et al*. (2009), investigating the effect of transfection with a plasmid encoding shRNA against the hTERT gene on ovarian cancer cells, showed that silencing of this gene results in cell cycle arrest in S-phase^31^.

In this paper, an increase in the percentage of FaDu cells in G1 phase and a decrease in the fraction of cells in G2 phase were noticed subsequent to siRNA/shRNA mediated knockdown. In the case of the H103, an increase in the fraction of cells in the S and G2 phases along with a decrease in the G1 phase was shown. These results indicate that, depending on the cell line, reduced hTERT expression results in cell cycle arrest either in phase G1 or S/G2. Regardless, where the cell cycle is stopped is where all cells are ultimately directed onto the apoptosis pathway.

Moreover, the cell cycle analysis by flow cytometry allowed for additional confirmation of the induction of apoptosis following hTERT gene silencing (siRNA). In both cell lines, a significant increase in apoptotic sub-G1 cell fraction compared with control was observed. Shammas *et al*. (2005) in esophageal cancer and Dong *et al*. (2009) in the breast cancer model reported a similar increase in apoptotic cell fraction^27,32^. Gandellini *et al*. (2007) in the study of the effect of hTERT gene silencing on the induction of apoptosis in prostate cancer cells observed only a slight increase in this fraction^16^. Such an effect was not reported for pancreatic cancer^17^.

According to literature data, production of DSBs is directly connected not only with cell cycle arrest but also as a result of exposure to ionizing radiation^33^.

We demonstrated that hTERT knockdown (with shRNA) in both studied^34^ cell lines resulted in an increase of DSBs compared to control. Such an effect was also detected after irradiation with 0.5 Gy and 1 Gy doses in FaDu cells. Increased sensitivity to ionizing radiation with dose of 2 Gy in H103 hTERT gene knockdown cells was demonstrated. Measurement of the level of DSBs following irradiation of cells with silenced hTERT gene with simultaneous administration of cytostatics (cisplatin and docetaxel) was also performed. After the administration of cisplatin and exposure to radiation (0.5 TPL and dose of 0.5 Gy and 1 Gy, and 1 TPL and dose of 2 Gy), an increase in DSBs level was noticed in both cell lines. With docetaxel, an elevated DSBs level was observed in the H103 cell line. Concomitant use of hTERT gene expression depletion and administration of docetaxel effectively inhibited proliferation of FaDu cells, thus undermining successful DSBs analysis. In the case of using siRNA-based strategy in FaDu cells, irradiation (1 and 2 Gy) led to an increase in phosphorylation of the γH2AX marker compared to control on day 3. On day 7, the DSBs level was not dependent on radiation dose. A similar phenotype was observed in H103 cells.

The results obtained in the study confirm the observations made by Takahashi *et al*. (2014). Using therapy with telomerase-specific oncolytic adenovirus OBP-301, the authors demonstrated enhanced response to radiation in laryngeal, tongue, and pharyngeal cancer *in vitro*^5^. A correlation between reduced telomerase activity and efficient response to radiotherapy was also demonstrated by Ogawa *et al*. (1998)^34^. Observations made in this study are also supported by experiments carried out on different cancer models. Similar results were obtained by Chen *et al*. (2012), using siRNAs directed against the hTR subunit in cervical cancer^35^. The magnification of response to radiotherapy has also been reported in the esophageal cancer^36^ and lung cancer models^37^. The results obtained in this work suggest that hTERT gene silencing results in an increased level of DSBs, which can be further magnified by simultaneous administration of ionizing radiation and/or cytostatics.

The demonstrated effect of hTERT gene silencing on DSBs production, cell cycle arrest, and apoptosis induction suggests the possibility of activation of DNA damage repair mechanisms. Up to date, literature data only describe cell cycle arrest impact on activation of individual DDR mechanisms^38^. There are still no sufficient data concerning the effect of the hTERT gene silencing using RNA interference on the activation of these mechanisms in cancer cells. This is the first time when such an analysis has been performed.

Significant increase in expression of CSB and XPA genes demonstrating activation of the NER mechanism have been reported in both FaDu and H103 cell lines. Elevated expression of the MSH6 gene (accounting for MMR mechanism) at the transcription level and an increase in XRCC4 gene expression (NHEJ mechanism) at the protein level in the FaDu cell line was also observed. Increased expression of the XRCC1 (BER) and PRKDC (NHEJ) genes on day 7 was also shown in H103 cells. The results suggest that hTERT silencing results in activation of NER as the main mechanism of DNA repair. Therefore, it can be assumed that the inhibition of activated DDR mechanisms in combination with the knockdown of the hTERT gene by RNA interference can strengthen the therapeutic effect.

## Conclusions

Results reported in this study indicate that a combined approach (chemotherapy, radiotherapy, gene therapy) may be significantly beneficial in reducing chemotherapy doses, thus shortening the hospitalization period and improving patients’ quality of life.

## Materials and Methods

### Plasmids construction

shRNA constructs were created by annealing of designed complementary oligonucleotides (sense: AATTCCCGAACACCAAGAAGTTCATCTTCAAGAGAGATGAACTTCTTGGTGTTCTTTTTG; antisense: CGCGCAAAAAGAACACCAAGAAGTTCATCTCTCTTGAAGATGAACTTCTTGGTGTTCGGG) (containing EcoRI and MluI sites) and inserting them into the EcoRI and MluI sites of pLV-THEM-GP1 vector as previously described^39^. All DNA constructs were verified using a controlled digestion with EcoRI and XbaI enzymes (both ThermoFisher Scientific, MA, USA). The shRNAs were synthesized by Sigma Aldrich (MI, USA).

### Lentiviral vector production, transduction and titration

Lentiviral vector plasmids encoding the designed hairpins against hTERT gene were derived by cloning into pLV-THEM-GP1 lentiviral vector, which expresses the GFP gene under control of the H1 promoter (based on plasmid #12247; Addgene; Cambridge, MA, USA). Lentiviral vectors production, transduction and titration were performed according to the protocols described previously^39,40^. The vector was produced by using of 2^nd^ generation system, and co-transfection of lentiviral vector plasmid (pLV-THEM-shTERT), and packaging plasmids psPAX2 (Plasmid #12260, Addgene) and pMD2.G (Plasmid #12259, Addgene) with CaCl_2_. After four days, the lentiviral vector containing supernatant was collected, filtered (0.45 μm), concentrated, and aliquots were stored at −80 °C as previously described^39^. To assess the viral copy number at an integrated DNA level, the lentiviral vector titer was measured via SYBR green-based (Roche, Germany) real-time qPCR by means of WPRE (Woodchuck Hepatitis Virus Posttranscriptional Regulatory Element) method and albumin genes as templates, as described previously^40^.

### Transfection

Transfection of siRNA (siTERT) (sense: GAACACCAAGAAGUUCAUC[dT][dT]; antisense: GAUGAACUUCUUGGUGUUC[dT][dT]) was performed according to the manufacturer protocol using Lipofectamine® RNAiMAX (Thermo Scientific, MA, USA). As a negative control a non-specific dsRNAs conjugated with fluorescein, dsRNA BLOCK-iT™ Fluorescent Oligo (Thermo Scientific, MA, USA) were used.

### Cell Culture

In order to verify the specificity of hTERT downregulation in different cell types with basal telomerase activity, two different cancer cell lines were cultured, i.e. p53 mutant HNSCC cell lines FaDu (kindly obtained from Prof. Michael Baumann, (OncoRay - National Center for Radiation Research in Oncology, Technische Universität Dresden) and H103 (ECACC, 06092001). FaDu cells were cultured in DMEM (Biowest, France) medium supplemented with 10% FBS (Biowest, France) and 1% penicillin/streptomycin in an incubator at 37 °C, 5% CO_2_ atmosphere and a humidity level of 100%. H103 cells were cultured in DMEM/HAM (1:1) (Biowest, France) medium supplemented with 10% FBS, 1% penicillin/streptomycin and 1% L-glutamine (all supplements from Biowest, France) in the same conditions as FaDu cell line.

### Cell migration assay *in vitro*

Cell migration was evaluated using the wound healing assay^41^. Briefly, FaDu and H103 cells were plated in a 12-well plate at a concentration of 0.2 × 10^6^ cells/well, transfected and allowed to form a confluent monolayer for 72 hours. Prior to wounding, cells were starved in serum-free medium for 20 hours. Next, a 200 µl pipette tip was used to create the wound. Cell migration was observed by microscopy (plates suited in racks in exactly the same positions [coordinates]) for up to 96 h in 24-h intervals and analyzed using ImageJ (National Institutes of Health, NY, USA). The percentage of wound closure was calculated using the following formula:

$$C=\frac{A(0)-A(t)}{A(0)}\times 100 \% $$

where: A(0) is the initial wound area, A(t) is the wound area at indicated time t.

### Real-Time PCR analysis

#### Assessment of individual genes expression

Quantitative analysis of gene expression was assessed using qPCR. Briefly, total RNA was extracted with TRI Reagent according to the manufacturer protocol (Sigma-Aldrich, MI, USA)^42^. cDNA was synthesized with iScript cDNA Synthesis Kit (Bio-Rad, CA, USA), using 500 ng of total RNA, oligo dT primers, and random hexamer primers. The real-time polymerase chain reaction for individual gene expression analysis was carried out using LightCycler 96 with specific primers (Table 1) designed with Universal Probe Library software (Roche Diagnostics, Switzerland). Amplification products of individual gene transcripts were detected with fluorescent probes (Universal Probe Library, Roche, Germany) and FastStart Essential Probes Master (Roche, Germany). The reaction conditions for all amplicons were as follows: initially 95 °C for 10 min, followed by 45 cycles at 95 °C for 10 s, 60 °C for 30 s, and 72 °C for 1 s. All reactions were performed in the presence of 3.2 mM MgCl_2_. The expression was normalized to the GAPDH housekeeping gene (Universal Probe Library Human GAPD Gene Assay, Roche, Germany) expression.

**Table 1:** List of primers and probes.

Gene	Primer forward	Primer reverse	Probe
hTERT	TGCAAAGCATTGGAATCAGA	ATGCTGCCTGACCTCTGC	#24
BECN1	ATGCAGGTGAGCTTCGTGT	GCCTGGGCTGTGGTAAGTAA	#71
CASP3	TTGTGGAATTGATGCGTGAT	GGCTCAGAAGCACACAAACA	#68
CASP9	TCAGGCCCCATATGATCG	GACTCCCTCGAGTCTCCAGAT	#42
ANXA5	GCAACTACTCCTTGCTGTTGTG	CCTGGAAACCATGACTCTGAT	#33
CDK2	AAAGCCAGAAACAAGTTGACG	GTACTGGGCACACCCTCAGT	#77
CDK4	AGCCAGAGAACATTCTGGTGA	CGGTACCAGAGTGTAACAACCA	#31
CCNA1	TCAGTACCTTAGGGAAGCTGAAA	CCAGTCCACCAGAATCGTG	#71
CCND1	GCTGTGCATCTACACCGACA	GCCAGGTTCCACTTGAGC	#68
p27/Kip1	TTTGACTTGCATGAAGAGAAGC	AGCTGTCTCTGAAAGGGACATT	#60
p34(Cdc2)	GGATTTCCTTCTTAGGTCACTGAA	TTTTTCTAAATGCGTGATTTGC	#36
XPA	CGAGTATCGAGCGGAAGC	TTACATTAGCCATGCCTCCA	#71
CSB	AAAGCATCTCCAGGCCATC	CATGCTGCCAAGACTGGAT	#68
APEX	GCTTCGAGCCTGGATTAAGA	TTGGTCTCTTGAAGGCACAGT	#45
XRCC1	CTGGGACCGGGTCAAAAT	CAAGCCAAAGGGGGAGTC	#71
XRCC4	TGGTGAACTGAGAAAAGCATTG	TGAAGGAACCAAGTCTGAATGA	#68
PRKDC	AGAGGCTGGGAGCATCACT	CACCAAGGCTTCAAACACAA	#31
MSH6	AATGACATTCTAATAGGCTGTGAGG	AACCCATCTGGGCCATTAC	#68
MSH2	GAGCCCTTAACCTTTTTCAGG	TTGTCCTTGAGGGGTTTTACAC	#71

### Telomere length assessment

DNA was extracted from cancer cells using Wizard Genomic DNA Purification Kit (Promega, WI, USA) according to manufacturer’s protocol, and stored at −20 °C. Telomere length was assessed using two pairs of primers i.e. telomere-specific and a single copy gene-specific (albumin), as previously described^43–45^. Briefly, we used the primers that were already shown to work specifically with conditions providing efficiency close to 100%. Initial denaturation and polymerase activation (hot start) was performed in 95 °C for 5 min. The signal was detected during 45 cycles i.e. 95 °C/10 s, 60 °C/15 s and 72 °C/10 s. Melting analysis (65–95 °C range, 0.11 °C resolution) at the end of the reaction melting analysis was performed to verify specificity of the product. The telomere length was assessed using a LightCycler 96 qPCR system (Roche, Germany) and FastStart Sybr Green Master (Roche, Germany).

### Immunofluorescence staining

Briefly, harvested cancer cells were fixed in 100% methanol at −20 °C for 20 min and washed three times with PBS (Biowest, France). After blocking by 1% BSA (Sigma-Aldrich, MI, USA) for 40 min in room temperature, cells were incubated with the primary antibody diluted in 1% BSA/PBS overnight at 4 °C. After being washed three times with PBS, cells were incubated with secondary antibody (1:500 in 1% BSA/PBS) for 1 h in the dark at 37 °C. After being washed three times with PBS, cells were incubated with 1:10,000 DAPI/H_2_O (Sigma-Aldrich, MI, USA) and were observed under fluorescence microscope (Leica, Germany) at room temperature. Primary antibodies were: 1:1000 anti-CASP3 rabbit polyclonal antibody (Abcam, UK), 1:750 anti-BECN1 mouse monoclonal antibody (Abcam, UK), and 1:500 anti-hTERT mouse monoclonal antibody (Abcam, UK). Secondary antibodies were: Alexa Fluor 488 anti-mouse IgM antibody, Alexa Fluor 594 anti-mouse IgM antibody (both antibodies from Jackson ImmunoResearch, PA, USA). Results were analyzed using reverse fluorescent microscope Opta-Tech MW-100 FL and presented as semi-quantitative using ImageJ software (National Institutes of Health, NY, USA).

### Flow cytometry

#### Cell Cycle

Cell cycle analysis was performed as previously described^46^. Briefly, after treatment with lentiviral vectors targeting hTERT and empty vector, cells were washed twice with 1 ml of PBS and fixed with 70% ethanol overnight at −20 °C. After washing twice with PBS, cells were centrifuged at 3000 rpm and 200 µl of a solution containing 250 μg/ml propidium iodide (Cayman Chemical, USA), 500 μg/ml RNAse (AppliChem, Germany) in PBS was added to the pellet, and incubated for 30 min at 37 °C in the dark. Data acquisition was performed with a BD Accuri C6 Plus (BD Biosciences, NJ, USA), and results were analyzed with FlowJo software (FlowJo, LLC, OR, USA).

### Apoptosis analysis

The analysis was performed according to the manufacturer protocol with FITC Annexin V Apoptosis Detection Kit I (BD Biosciences, NJ, USA). Briefly, cells were washed twice with PBS and stained by 5 µl Annexin V FITC-conjugated antibody and 5 µl propidium iodide. That approach, cross-staining with Annexin V and propidium iodide, allowed detection not only apoptosis (Annexin-positive cells), but also necrosis (propidium iodide-positive cells). After 15 min incubation in the dark, data were acquired with a BD Accuri C6 Plus flow cytometer (BD Biosciences, NJ, USA), and the results were analyzed using FlowJo software (FlowJo, LLC, OR, USA).

### Analysis of DNA Double-Strand Breaks

Cells were irradiated with 0.5 Gy, 1 Gy or 2 Gy doses with Gammacell® 1000 Elite (BestTheratronics Ltd, Canada). For the analysis of γH2AX status (DNA Double-Strand Breaks marker), cancer cells were prepared according to manufacturer protocol (Apoptosis, DNA Damage and Cell Proliferation Kit; Becton Dickinson, NJ, USA). Briefly, HNSCC cells were fixed with BD Cytofix/Cytoperm Fixation/Permeabilization Solution for 30 min in room temperature. Subsequently, cells were incubated with BD Cytofix/Cytoperm Plus Permeabilization Buffer for 10 min at 4 °C, and re-fixed, with BD Cytofix/Cytoperm Fixation/Permeabilization Solution (5 min, room temperature). After washing, cells were incubated with fluorochrome-conjugated Alexa Fluor 647 Mouse Anti-H2AX antibody (pS139). Staining with isotype control (APC Mouse IgG2b κ Isotype Control; Becton Dickinson, USA) antibodies were performed to assess the threshold of positive staining for specific antibody. Data acquisition was performed with a BD Accuri C6 Plus flow cytometer (BD Biosciences, NJ, USA), and results were analyzed using FlowJo software (FlowJo, LLC, OR, USA).

### Western blot analysis

Total protein lysates for Western blot analysis were extracted with RIPA buffer (SigmaAldrich, MO, USA). The concentration of protein in the sample was measured using Pierce™ BCA Protein Assay Kit (Thermo Scientific, MA, USA) according to the manufacturer protocol. 10 µg of total protein of each cell extract was resolved in Tris/Glycine/Sodium dodecyl sulphate polyacrylamide gel electrophoresis (Bio-Rad Laboratories Ltd, CA, USA) and transferred to a polyvinylidinedifluoride membrane (Trans-Blot® Turbo™ Transfer Pack (Bio-Rad Laboratories Ltd, CA, USA). Nonspecific binding was blocked by incubation in 5% non-fat milk in Tris-buffered saline and Tween 20 at room temperature for 1 h. Blots were then probed overnight at 4 °C with anti-hTERT (1:1000, Rockland, Germany), anti-CASP3 (1:1000, Abcam, England), anti-BECN1 (1:750, Abcam, England), anti-CSB (1:100, Abcam, England), anti-MSH2 (1:1000, Abcam, England), anti-XRCC1 (1:1000, Abcam, England), anti-XRCC4 (1:1000, Abcam, England), and anti-β-AKT (Santa Cruz Biotechnology, CA, USA) antibodies. Immunoreactive bands were then probed for 1 h at room temperature with the appropriate horseradish peroxidase (HRP)-conjugated secondary anti-Rabbit IgG-HRP (1:2000, Cell Signaling Technology, MA, USA) antibody. Protein bands were detected by WesternBright™ Quantum reagent (Advansta, CA, USA) and analysed using a ChemiDoc Touch Imaging System (Bio-Rad Laboratories Ltd, CA, USA). Additionally, results were analyzed semi-quantitative using ImageJ software (National Institutes of Health, NY, USA).

### Cytostatics

Cytostatic drugs – cisplatin (Teva Pharmaceuticals, Poland), docetaxel (Actavis, Iceland) – were used in this study at tolerable plasma level (TPL) (6.667 μM for cisplatin and 550 nM for docetaxel).

### Statistical analysis

Two-tailed unpaired Student’s t test, ANOVA, and Chi-squared test (means from 3 separate experiments) were performed for statistical analysis using GraphPad Prism (GraphPad Software, CA, USA). P values of less than 0.05 were considered statistically significant and are indicated by the (*) symbol for p < 0.05, by (**) for p < 0.01, by (***) for p < 0.001, or by (****) for p ≤ 0.0001.

### Data availability statement

All data generated or analyzed during this study are included in this published article.
